# Exploring the influence of behavioral factors on depression and anxiety scores during the COVID-19 pandemic: insights from the Virginia statewide COVIDsmart longitudinal study

**DOI:** 10.1186/s12889-023-16614-7

**Published:** 2023-09-08

**Authors:** Matvey V. Karpov, Marilyn M. Bartholmae, Brian L. Levy, Amira A. Roess, Keith D. Renshaw, Joshua M. Sill, Sunita Dodani

**Affiliations:** 1 Eastern Virginia Medical School-Sentara Healthcare Analytics and Delivery Science Institute, Norfolk, VA USA; 2 Department of Psychiatry and Behavioral Sciences, Eastern Virginia Medical School, Norfolk, VA USA; 3 Department of Sociology & Anthropology, George Mason University, Fairfax, VA USA; 4 Department of Global and Community Health, George Mason University, Fairfax, VA USA; 5 Department of Psychology, George Mason University, Fairfax, VA USA; 6 Division of Pulmonology, Department of Internal Medicine, Eastern Virginia Medical School, Norfolk, VA USA; 7 Division of Cardiology, Department of Internal Medicine, Eastern Virginia Medical School, Norfolk, VA USA

**Keywords:** COVID-19, Mental health, Health behaviors, Tobacco, Smoking, Alcohol, Physical activity, Social media, Loneliness, Health promotion

## Abstract

**Background:**

Amidst the COVID-19 pandemic, there has been growing concern about the declining mental health and healthy behaviors compared to pre-pandemic levels. Despite this, there is a lack of longitudinal studies that have examined the relationship between health behaviors and mental health during the pandemic. In response, the statewide COVIDsmart longitudinal study was launched. The study’s main objective is to better understand the effects of the pandemic on mental health. Findings may provide a foundation for the identification of public health strategies to mitigate future negative impacts of the pandemic.

**Methods:**

Following online recruitment in spring of 2021, adults, ages 18 to 87, filled out social, mental, economic, occupational, and physical health questionnaires on the digital COVIDsmart platform at baseline and through six monthly follow-ups. Changes in the participant’s four health behaviors (e.g., tobacco and alcohol consumption, physical activity, and social media use), along with sex, age, loneliness score, and reported social and economic (SE) hardships, were analyzed for within-between group associations with depression and anxiety scores using Mixed Models Repeated Measures.

**Results:**

In this study, of the 669 individuals who reported, the within-between group analysis indicated that younger adults (F = 23.81, *p* < 0.0001), loneliness (F = 234.60, *p* < 0.0001), SE hardships (F = 31.25, *p* < 0.0001), increased tobacco use (F = 3.05, *p* = 0.036), decreased physical activity (F = 6.88, *p* = 0.0002), and both positive and negative changes in social media use (F = 7.22, *p* = 0.0001) were significantly associated with worse depression scores. Additionally, females (F = 6.01, *p* = 0.015), younger adults (F = 32.30, *p* < 0.0001), loneliness (F = 154.59, *p* < 0.0001), SE hardships (F = 22.13, *p* < 0.0001), increased tobacco use (F = 4.87, *p* = 0.004), and both positive and negative changes in social media use (F = 3.51, *p* = 0.016) were significantly associated with worse anxiety scores. However, no significant changes were observed in the within-between group measurements of depression and anxiety scores over time (*p* > 0.05). Physical activity was not associated with anxiety nor was alcohol consumption with both depression and anxiety (*p* > 0.05).

**Conclusions:**

This study demonstrates the longitudinal changes in behaviors within the context of the COVID-19 pandemic. These findings may facilitate the design of preventative population-based health approaches during the COVID-19 pandemic or future pandemics.

**Supplementary Information:**

The online version contains supplementary material available at 10.1186/s12889-023-16614-7.

## Background

Numerous studies have demonstrated that COVID-19 has had a negative impact on mental health and has led to increased psychological distress such as anxiety and depression compared to pre-pandemic levels [1–3]. There is also a growing body of literature on the relationship between health behaviors and depression and anxiety [4]. Exercise, especially high intensity training, is one of the best health behaviors an individual can adopt to improve physical and mental health [5]. Previous studies reported that an increase in alcohol or tobacco consumptions, as well as a decrease in physical activity, have had a negative impact on mental health during the pandemic [6, 7]. Research on the impact of social media usage on mental health show mixed results, some longitudinal studies reported social media usage as a non-factor, a risk factor, or a protective factor [8–10]. During the pandemic, many researchers turned to digital research methods to continue conducting research while mitigating risk of infection. This includes studies of the impacts of COVID-19 on health outcomes [11, 12]. Along with focusing on health safety, digital studies facilitate recruitment of large sample of participants, especially those that live in rural areas [13]. They also reduce the cost and participant burden for collecting multiple waves of data over a short time period.

Using digital research methods, the COVIDsmart longitudinal study aimed to evaluate the relationship between COVID-19-related health behaviors and the mental health of individuals in Virginia. By examining this relationship, this study sought to shed light on the significance of personalized health promotion initiatives during a pandemic.

## Methods

COVIDsmart was an online statewide study developed in collaboration with Eastern Virginia Medical School-Sentara Healthcare Analytics and Delivery Science Institute, George Mason University, and Vibrent Health Inc. Data were collected using an online platform in compliance with the Health Insurance Portability and Accountability Act (HIPAA) [14, 15]. Participants received email invitations to join the COVIDsmart platform and complete a series of questionnaires. Demographic data was collected at baseline, and participants’ personal experiences impacted by COVID-19, mental health and behaviors, and COVID-related occupational experiences were collected at baseline and at each of the six monthly follow-ups [16, 17]. Specifically, the present study evaluated the participant’s sex, age, a sum of the social and economic (SE) hardships experienced (i.e., lost income from a job or business, job loss, unable to get groceries, etc.) (Appendix A) [18], as well as validated measures of depression (using the Patient Health Questionnaire-9 or PHQ-9) [19], anxiety (using the Generalized Anxiety Disorder-7 or GAD-7), [20] and loneliness (using a shortened version of the University of California Los Angeles Loneliness scale) [21]. Additionally, the participants’ changes in health behaviors such as alcohol and tobacco consumption, physical activity, and social media use (such as Facebook or Twitter) were also evaluated. Participants were asked whether their behaviors have increased, decreased, approximately stayed the same, or were not applicable (N/A) in the past two weeks (Appendix B).

### Study participants

Recruitment strategies and processes have been detailed by Schilling et al. [14] and Bartholmae et al. [15]. Briefly, marketing tools such as radio, television, emails, newsletters, and social media were employed to invite residents of Virginia. Recruitment occurred from March to May 2021, and data was collected from March to November 2021. Inclusion criteria included being a resident of Virginia, United States, between the 18 and 87 years of age, proficient in English, and had access to the internet via a personal email account or a mobile phone number. Eligible participants were sent a digital consent form to join the study. A total of 782 participants (*N* = 782) gave informed consent to participate in the study. To encourage continuous engagement, participants who completed the questionnaires at each of the six monthly follow-ups had a 1 in 20 chance of winning a $50 gift card each month. At the end of the study, active participants had a 1 in 4 chance of winning a $500 gift card [15].

### Statistical analysis

The study conducted descriptive statistics on several factors such as participants' demographics, health behaviors, loneliness, reported number of SE hardships, and their depression (PHQ-9) and anxiety (GAD-7) scores. Mixed Models Repeated Measures (MMRM) were used to evaluate the changes in behaviors over the six months and their association with the outcomes, depression and anxiety. The demographics collected at baseline were included in the MMRM as covariates. Independent variables, (loneliness, SE hardships, and heath behaviors) and dependent variables (depression and anxiety) included in the MMRM were collected at baseline and follow-ups one through six. Within-between group comparisons were evaluated across all time points of the study. Additional bivariate analyses were conducted between significant independent variables from the MMRM analyses at each time point of the study. Mann–Whitney U tests were used to measure the differences between males’ and females' depression and anxiety scores. Spearman's correlation was used to measure the association between age, the loneliness scale score, and the number of SE hardships with depression and anxiety scores. Kruskal–Wallis tests were used to measure the difference in the depression and anxiety scores among participants who reported changes in health behaviors such as alcohol, tobacco, physical activity, and social media in the past two weeks. Post-hoc tests were conducted using the Dwass, Steel, Critchlow-Fligner (DSCF) method in cases where the Kruskal–Wallis test found a significant difference in mental health outcome among the health behaviors. Statistical analyses were performed using SAS version 9.4, and *p*-values less than 0.05 were considered significant.

## Results

### Demographics

Out of the 782 participants who consented to be in the study, 669 participants have completed the questionnaires on their mental health at baseline in the COVIDsmart study. Of these participants’ demographics described in Table 1, most were female (78.30%), middle aged (µ = 50.54, SD = 14.44), non-Hispanic White (94.70%), have earned a post-secondary degree (80.88%), and annually earn at least $50,000 (88.20%). Most participants lived in a two-person household (37.70%) and lived with their married or unmarried partner (68.90%).

**Table 1:** Baseline demographics and health behaviors of the COVIDsmart participants throughout the study

	Baseline (*n* = 669, 100%)	Follow-up 1 (*n* = 444, 66.40%)	Follow-up 2 (*n* = 359, 53.70%)	Follow-up 3 (*n* = 298, 44.50%)	Follow-up 4 (*n* = 249, 37.20%)	Follow-up 5 (*n* = 236, 35.30%)	Follow-up 6 (*n* = 212, 31.70%)	*p*-value
Demographics n (%)								
Sex (*n* = 667)								0.997
Male	145 (21.70)	91 (20.60)	77 (21.50)	63 (21.10)	55 (22.10)	53 (22.50)	48 (22.60)	
Female	522 (78.30)	351 (79.40)	281 (78.50)	235 (78.90)	194 (77.90)	183 (77.50)	164 (77.40)	
Age (*n* = 669)								** < 0.0001**
μ (SD)	50.54 (14.44)	50.73 (14.87)	51.78 (15.20)	53.22 (15.03)	54.83 (14.75)	55.46 (14.69)	55.76 (14.89)	
Race (*n* = 663)								0.997
White	592 (89.30)	397 (90.90)	317 (90.10)	265 (90.80)	224 (91.80)	210 (90.90)	190 (91.80)	
African American	44 (6.70)	23 (5.20)	20 (5.70)	16 (5.40)	13 (5.30)	13 (5.60)	10 (4.80)	
Other	23 (3.50)	17 (3.90)	15 (4.20)	11 (3.80)	7 (2.90)	8 (3.50)	7 (3.40)	
Ethnicity (*n* = 665)								0.206
Hispanic	35 (5.30)	20 (4.50)	14 (3.90)	12 (4.00)	6 (2.40)	5 (2.10)	5 (2.40)	
Non-Hispanic	630 (94.70)	424 (95.50)	345 (96.10)	286 (96.00)	243 (97.60)	231 (97.90)	207 (97.60)	
Education Level (*n* = 662)								0.994
No high school diploma	2 (0.30)	2 (0.50)	2 (0.60)	2 (0.70)	0	0	0	
High school diploma or GED	30 (4.50)	19 (4.30)	17 (4.80)	16 (5.40)	14 (5.70)	14 (6.00)	14 (6.60)	
Some college	93 (14.00)	60 (13.60)	38 (10.70)	32 (10.80)	28 (11.30)	28 (11.90)	25 (11.80)	
Associate’s degree	53 (8.00)	27 (6.10)	20 (5.60)	21 (7.10)	17 (6.90)	17 (7.20)	16 (7.60)	
Bachelor's degree	208 (31.40)	144 (32.70)	123 (34.60)	91 (30.70)	73 (29.60)	68 (28.90)	60 (28.30)	
Master's degree	212 (32.00)	144 (32.70)	120 (33.70)	105 (35.50)	92 (37.30)	86 (36.60)	77 (36.30)	
Doctoral or Professional degree	64 (9.70)	45 (10.20)	36 (10.10)	29 (9.80)	23 (9.30)	22 (9.40)	20 (9.40)	
Income level (*n* = 667)								0.999
< $50,000	79 (11.80)	51 (11.60)	41 (11.50)	28 (9.50)	23 (9.30)	22 (9.40)	18 (8.50)	
$50,000—$99,999	185 (27.70)	119 (26.90)	105 (29.40)	93 (31.40)	72 (29.00)	70 (29.80)	61 (28.90)	
$100,000—$149,999	164 (24.60)	116 (26.20)	96 (26.90)	80 (27.00)	69 (27.80)	63 (26.80)	58 (27.50)	
$150,000 +	196 (29.40)	130 (29.40)	93 (26.10)	77 (26.00)	68 (27.40)	65 (27.70)	59 (28.00)	
Prefer not to answer	43 (6.50)	26 (5.90)	22 (6.10)	18 (6.10)	16 (6.50)	15 (6.30)	15 (7.10)	
Household size (*n* = 669)								0.202
1	112 (16.70)	85 (19.10)	77 (21.50)	66 (22.10)	55 (22.10)	57 (24.10)	54 (25.50)	
2	252 (37.70)	174 (39.20)	145 (40.40)	120 (40.30)	103 (41.40)	96 (40.70)	89 (42.00)	
3	136 (20.30)	80 (18.00)	58 (16.10)	53 (17.80)	42 (16.90)	37 (15.70)	30 (14.10)	
4	103 (15.40)	68 (15.30)	51 (14.20)	35 (11.70)	25 (10.00)	24 (10.10)	19 (9.00)	
5 +	66 (9.90)	37 (8.30)	28 (7.80)	24 (8.10)	24 (9.60)	22 (9.30)	20 (9.30)	
Marital status (*n* = 665)								0.999
Single	101 (15.20)	71 (16.10)	61 (17.10)	52 (17.60)	43 (17.50)	41 (17.50)	38 (18.10)	
Married or unmarried couple	458 (68.90)	298 (67.60)	232 (653.20)	191 (64.50)	161 (65.20)	150 (64.10)	133 (63.30)	
Widowed	23 (3.50)	17 (3.90)	16 (4.50)	13 (4.40)	10 (4.00)	11 (4.70)	10 (4.80)	
Divorced or separated	83 (12.50)	55 (12.50)	47 (13.20)	40 (13.50)	33 (13.40)	32 (13.70)	29 (13.80)	
Changes in the past two weeks								
Tobacco use (*n* = 634)								**0.011**
Decreased	7 (1.10)	7 (1.70)	1 (0.30)	1 (0.40)	1 (0.40)	1 (0.40)	1 (0.50)	
Approximately the same	47 (7.40)	38 (9.20)	28 (8.40)	17 (6.10)	13 (5.50)	15 (6.50)	10 (4.90)	
Increased	27 (4.30)	5 (1.20)	4 (1.20)	3 (1.00)	5 (2.10)	2 (0.90)	4 (1.90)	
N/A	553 (87.20)	363 (87.90)	302 (90.10)	260 (92.50)	219 (92.00)	214 (92.20)	191 (92.70)	
Alcohol consumption (*n* = 664)								** < 0.0001**
Decreased	88 (13.30)	60 (13.70)	53 (15.00)	43 (14.70)	29 (11.80)	28 (12.00)	26 (12.60)	
Approximately the same	295 (44.40)	236 (53.80)	195 (55.20)	160 (54.80)	139 (56.50)	125 (53.40)	113 (54.60)	
Increased	140 (21.10)	33 (7.50)	21 (6.00)	21 (7.20)	18 (7.30)	13 (5.60)	10 (4.80)	
N/A	141 (21.20)	110 (25.00)	84 (23.80)	68 (23.30)	60 (24.40)	68 (29.00)	58 (28.00)	
Physical activity (*n* = 599)								** < 0.0001**
Decreased	245 (40.90)	77 (19.80)	50 (16.30)	32 (12.40)	37 (16.20)	32 (14.70)	33 (16.80)	
Approximately the same	193 (32.20)	207 (53.20)	185 (60.20)	170 (65.90)	144 (62.90)	138 (63.30)	131 (66.50)	
Increased	156 (26.00)	103 (26.50)	69 (22.50)	55 (21.30)	46 (20.00)	46 (21.10)	32 (16.20)	
N/A	5 (0.80)	2 (0.50)	3 (1.00)	1 (0.40)	2 (0.90)	2 (0.90)	1 (0.50)	
Social media use (*n* = 578)								** < 0.0001**
Decreased	56 (9.70)	42 (11.60)	39 (13.90)	30 (12.50)	25 (11.40)	22 (10.40)	21 (10.80)	
Approximately the same	225 (38.90)	243 (67.30)	190 (67.90)	160 (66.30)	149 (68.00)	138 (65.40)	130 (67.00)	
Increased	271 (46.90)	50 (13.90)	27 (9.60)	25 (10.40)	19 (8.70)	19 (9.00)	18 (9.30)	
N/A	26 (4.50)	26 (7.20)	24 (8.60)	26 (10.80)	26 (11.90)	32 (15.20)	25 (12.90)	

Bold values indicate statistical significance at *p* < 0.05 level

Also described in Table 1, initially, 669 participants completed the questionnaires at baseline but, only 66.40% of the participants (*n* = 444) had reported at the first follow-up. Subsequently, monthly follow-up completion rates declined, with 53.70% (*n* = 359), 44.50% (*n* = 298), 37.20% (*n* = 249), and 35.30% (*n* = 236) completing follow-ups two, three, four, and five, respectively. At the final follow-up, 212 (31.70%) of the original 669 participants had completed all six follow-ups. A detailed table for screening and eligibility rates was published previously by Schilling et al. [14]. However, the proportion of most of the demographic groups remained relatively the same throughout the study, *p* > 0.05, except the mean age had increased at each follow-up (*p* < 0.0001) (Table 1).

#### Mixed models repeated measures

The MMRM (Table 2) estimated the impact of various demographic, behavioral, social, and economic factors on the depression (PHQ-9) scores of study participants over a period of time. The within-between group results demonstrated that age (F = 23.81, df = 1, *p* < 0.0001), COVID-19 related SE hardships (F = 31.25, df = 1, *p* < 0.0001), loneliness (F = 234.60, df = 1, *p* < 0.0001), tobacco use (F = 3.05, df = 3, p = 0.036), physical activity (F = 6.88, df = 3, *p* = 0.0002), and social media use (F = 7.22, df = 3, *p* = 0.001) were all significantly associated with depression scores. However, sex (F = 3.67, df = 1, *p* = 0.056), alcohol use (F = 1.18, df = 3, *p* = 0.316), and time (F = 0.98, df = 6, *p* = 0.435) did not significantly impact the participants’ depression scores. None of the within-between group comparisons over time were statistically significant (*p* > 0.05).

**Table 2:** Mixed models repeated measures of depression (PHQ-9) and anxiety (GAD-7) scores

	**PHQ-9**			**GAD-7**		
Effect	F-Value	df	*p-value*	F-Value	df	*p-value*
Sex	3.67	1	0.056	6.01	1	**0.015**
Age	23.81	1	** < 0.0001**	32.30	1	** < 0.0001**
Number of SE Hardships	31.25	1	** < 0.0001**	22.13	1	** < 0.0001**
Loneliness scale	234.60	1	** < 0.0001**	154.59	1	** < 0.0001**
Alcohol use in the past two weeks	1.18	3	0.316	1.32	3	0.268
Tobacco use in the past two weeks	3.05	3	**0.036**	4.87	3	**0.004**
Physical Activity in the past two weeks	6.88	3	**0.0002**	1.14	3	0.331
Social Media use in the past two weeks	7.22	3	**0.0001**	3.51	3	**0.016**
Time	0.98	6	0.435	0.72	6	0.637
Time^a^Sex	0.68	6	0.664	1.21	6	0.298
Time^a^Age	0.50	6	0.807	0.25	6	0.959
Time^a^SE Hardships	1.84	6	0.087	1.25	6	0.278
Time^a^Loneliness	1.79	6	0.098	1.17	6	0.322
Time^a^Alcohol use	1.19	18	0.261	0.69	18	0.824
Time^a^Tobacco use	0.63	18	0.876	1.41	18	0.118
Time^a^Physical Activity	1.48	18	0.087	1.50	18	0.080
Time^a^Social Media use	1.05	18	0.405	1.17	18	0.282

Bold values indicate statistical significance at *p* < 0.05 level

^a^Interactions are defined as the effect of individual independent variables with time on the outcome

Another MMRM (Table 2) estimates the impact of the same list of independent factors on the participants’ anxiety (GAD-7) scores over a period of time. The within-between group results demonstrated that sex (F = 6.01, df = 1, *p* = 0.015), age (F = 32.30, df = 1, *p* < 0.0001), COVID-19 related SE hardships (F = 22.13, df = 1, *p* < 0.0001), loneliness (F = 154.59, df = 1, *p* < 0.0001), tobacco (F = 3.51, df = 3, *p* = 0.016), and social media (F = 3.51, df = 3, *p* = 0.016) were all significantly associated with anxiety scores. However, alcohol (F = 1.32, df = 3, *p* = 0.268), physical activity (F = 1.14, df = 3, *p* = 0.331), and time (F = 1.14, df = 6, *p* = 0.331) did not impact the anxiety scores. Additionally, none of the within-group comparisons over time were found not to be statistically significant (*p* > 0.05).

#### Depression (PHQ-9)

Following the results of the MMRM, a series of Spearman’s correlations revealed that participant’s age had weak negative correlation to their depression (PHQ-9) scores at all time points (*p* < 0.0001). Similarly, Spearman’s correlations revealed that the participant’s loneliness was strongly, positively correlated with their depression scores at all time points (*p* < 0.0001). Additionally, the participant’s reported SE hardships were weakly to moderately, positively correlated with their depression scores at all time points (*p* < 0.0001) (Table 3).

**Table 3:** Spearman’s correlations between age, loneliness, and social and economic (SE) hardships and depression (PHQ-9) and anxiety (GAD-7) scores over time

	**Depression**			**Anxiety**		
	Age	Loneliness	SE hardships	Age	Loneliness	SE hardships
**Time**	r_s_	r_s_	r_s_	r_s_	r_s_	r_s_
Baseline (*n* = 669)	-0.288	0.565	0.368	-0.344	0.510	0.350
Follow-up 1 (*n* = 444)	-0.290	0.576	0.401	-0.321	0.523	0.369
Follow-up 2 (*n* = 359)	-0.298	0.607	0.402	-0.340	0.576	0.384
Follow-up 3 (*n* = 298)	-0.336	0.567	0.407	-0.318	0.544	0.390
Follow-up 4 (*n* = 249)	-0.354	0.648	0.282	-0.365	0.582	0.319
Follow-up 5 (*n* = 236)	-0.321	0.518	0.282	-0.382	0.463	0.290
Follow-up 6 (*n* = 212)	-0.332	0.576	0.315	-0.327	0.500	0.389
*p-value*	** < 0.0001**	** < 0.0001**	** < 0.0001**	** < 0.0001**	** < 0.0001**	** < 0.0001**

Bold values indicate statistical significance at *p* < 0.05 level

**Fig. 1:**
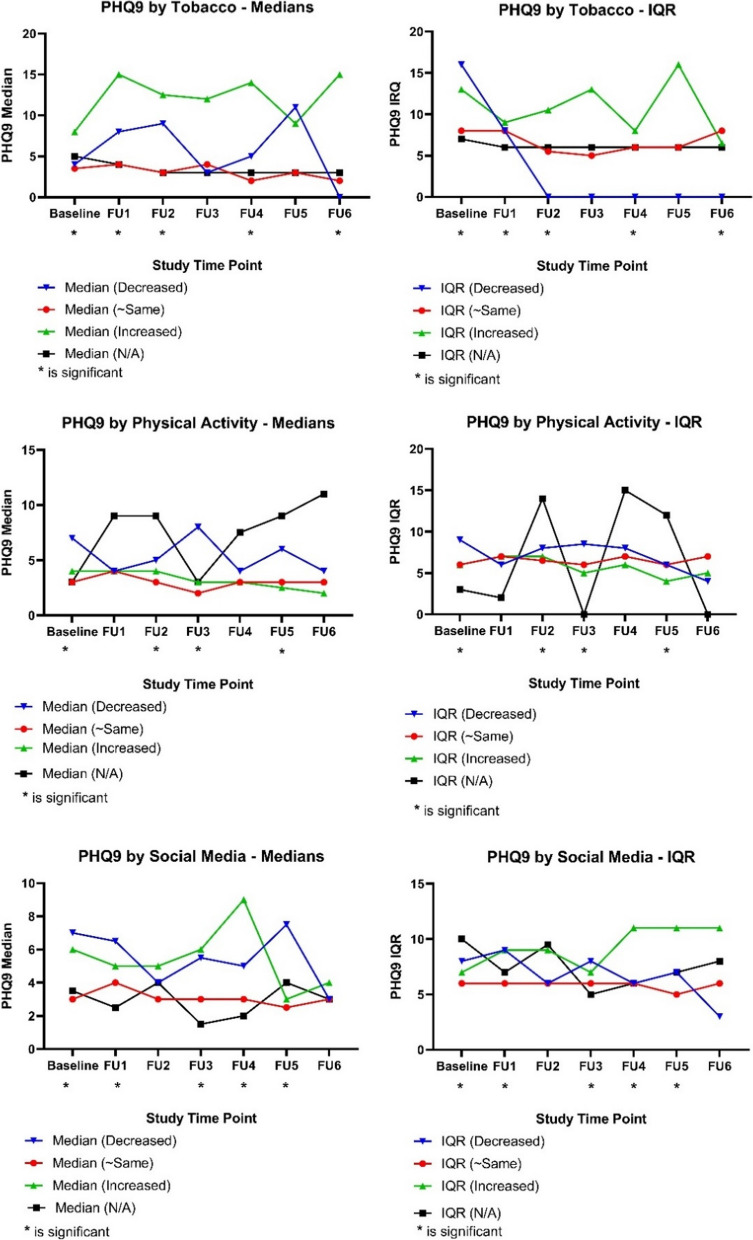
Medians and interquartile ranges (IQR) of depression (PHQ-9) scores by changes in tobacco use, physical activity, and social media use

The Kruskal–Wallis tests overall have found significant associations with changes in tobacco consumption and the participant’s depression (PHQ-9) scores at baseline (χ^2^ = 14.22, *p* = 0.003), follow-up one (χ^2^ = 10.78, *p* = 0.013), follow-up two (χ^2^ = 9.33, *p* = 0.025), follow-up four (χ^2^ = 8.95, *p* = 0.030), and follow-up six (χ^2^ = 11.44, *p* = 0.010). The following the post-hoc DSCF pairwise comparison also revealed that participants who reported an increase in tobacco consumption in the last two weeks also reported higher average depression compared to those who responded with approximately the same or “N/A” (Fig. 1).

Another series of Kruskal–Wallis tests indicated an association between the changes in the participant’s physical activity in the last two weeks and the depression (PHQ-9) scores at baseline (χ^2^ = 56.50, *p* < 0.0001), follow-up two (χ^2^ = 16.75, *p* = 0.001), follow-up three (χ^2^ = 25.22, *p* < 0.0001), and follow-up five (χ^2^ = 10.81, *p* = 0.013). The post hoc DSCF method also revealed that participants who reported decreased physical activity had higher depression scores compared to participants whose activity increased or remained approximately the same (Fig. 1).

Regarding the effect of social media and the participant’s depression (PHQ-9) scores, the Kruskal Wallis tests reported a significant association at baseline (χ^2^ = 35.00, *p* < 0.0001), follow-up one (χ^2^ = 14.40, *p* = 0.002), follow-up three (χ^2^ = 13.70, *p* = 0.003), follow-up four (χ^2^ = 16.55, *p* = 0.001), and follow-up five (χ^2^ = 12.38, *p* = 0.006). The DSCF post hoc tests indicated that participants who reported an increase or a decrease in social media use also reported higher depression scores compared to those who remained approximately the same. However, at some follow-ups, only those who responded with decreased social media use were associated with higher depression scores (Fig. 1).

#### Anxiety (GAD-7)

The Spearman’s correlations have revealed that age had a weak negative correlation with the participant’s anxiety (GAD-7) scores (*p* < 0.0001). Similarly, significant Spearman’s correlations have found that loneliness was positively, moderately correlated with the anxiety scores (*p* < 0.0001). And finally, COVID-19 related SE hardships were also significantly and positively, albeit weakly, correlated with the participant’s anxiety scores (*p* < 0.0001) Table 3.

**Fig. 2:**
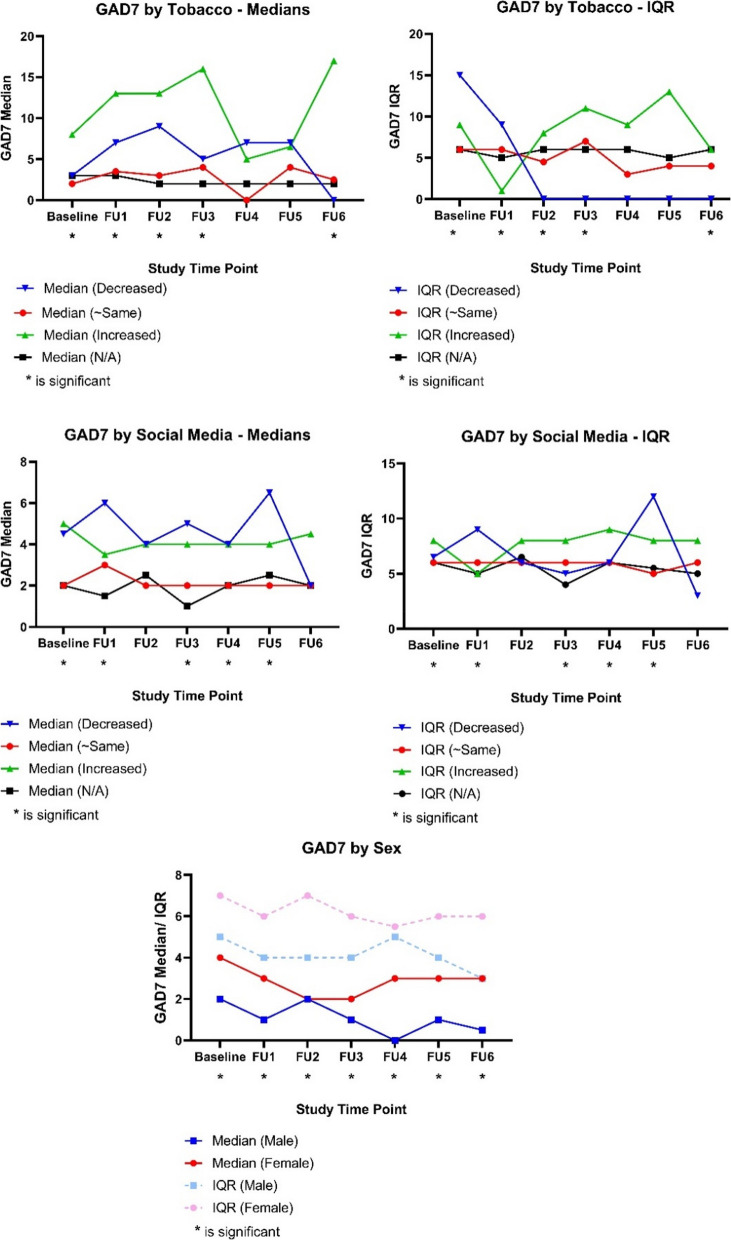
Medians and interquartile ranges (IQR) of anxiety (GAD-7) scores by sex and changes in tobacco and social media use

The participants who reported an increase in tobacco consumption in the last two weeks also reported higher average anxiety at baseline (χ^2^ = 21.23, *p* < 0.0001), follow-up one (χ^2^ = 13.01, *p* = 0.005), follow-up two (χ^2^ = 10.29, *p* = 0.016), follow-up three (χ^2^ = 8.50, *p* = 0.037), and follow-up six (χ^2^ = 11.23, *p* = 0.011). The post hoc DSCF test generally indicated that participants who reported increased tobacco consumption in the past two weeks had higher anxiety scores compared to those whose consumption remained approximately the same or responded with “N/A” (Fig. 2).

Regarding the effect of social media and the participant’s anxiety (GAD-7) scores, the Kruskal Wallis tests have found a significant association at baseline (χ^2^ = 33.79, *p* < 0.0001), follow-up one (χ^2^ = 11.90, *p* = 0.007), follow-up three (χ^2^ = 166.02, *p* = 0.001), follow-up four (χ^2^ = 9.81, *p* = 0.020), and follow-up five (χ^2^ = 12.53, *p* = 0.006). Similar to the models with depression (PHQ-9) scores, the DSCF post hoc tests also revealed that both participants who reported an increase or a decrease in social media use in the past two weeks had higher anxiety scores compared to participants who remained approximately the same. However, the reported associations were not consistent as at some time points, decreased social media use was linked with higher anxiety scores, whereas at other time points, only increased social media use indicated higher scores (Fig. 2).

Sex also played a role in the reported anxiety scores with the Mann–Whitney U tests have indicated that female participants reported significantly higher average anxiety scores than males at baseline (U = 38,395.50, *p* < 0.0001), follow-up one (U = 15,331.00, *p* < 0.0001), follow-up two (U = 11,108.50, *p* = 0.005), follow-up three (U = 7954.00, *p* = 0.018), follow-up four (U = 5364.00, *p* = 0.002), follow-up five (U = 5323.00, *p* = 0.026), and follow-up six (U = 3777.00, *p* = 0.001) (Fig. 2).

## Discussion

In this longitudinal study, we examined the effect of changes in alcohol consumption on mental health because alcohol is often used as a coping mechanism following a stressful event, despite its association with negative mental health [22]. However, the present study did not find a statistically significant association between changes in alcohol consumption and an increase in depression or anxiety over the course of the study. This may be due to the fact that majority of the COVIDsmart participants were well-educated white women with higher income, who, as reported by previous literature, are associated with lower rates of alcohol consumption and are therefore less likely to engage in heavy drinking or develop alcohol use disorders when coping with stressors [23–26]. For example, Probst et al. [23] reported in a meta-analysis that socioeconomically disadvantaged populations with low education and/or income were associated with higher relative risk of alcohol-attributable mortality. Higher socioeconomic factors, such as high income and higher educational attainment, as seen in this study, are examples of social determinants of health with protective factors against alcohol consumption.

Increased tobacco consumption was found to be significantly associated with higher levels of depression and anxiety. This finding is consistent with previous research, as Stanton et al. [7] also reported a positive association between an increase in tobacco consumption and a higher risk of depression and anxiety compared to those who reported no change or a decrease in tobacco consumption. These findings coincide with the American Lung Association and the Mental Health Foundation on the important implications for healthcare professionals and policymakers on the need to address the negative impact of tobacco use on mental health [27, 28]. It is essential to develop effective strategies to help individuals reduce or quit tobacco use, especially during the COVID-19 pandemic, where the negative impact of tobacco use on respiratory health may be compounded [29]. Additionally, these findings suggest that assessing an individual's tobacco use can provide insight into their risk of developing depression and anxiety. Therefore, it may be valuable to screen for tobacco use during mental health assessments.

In the current study, a decrease in physical activity was found to be associated with higher depression. These findings are similar to other studies the reported the positive effect of exercising on the mental well-being of an individual, including reducing symptoms of depression [7, 30, 31]. Specifically, a systematic review of reviews with meta-analysis published by Singh et al. [30] reported that physical activity reduced depression, especially accounting for the different types of exercise, session duration, and frequency per week. Different from current literature, this study did not find changes in physical activity to be associated with anxiety. Both Singh et al. [30] and Wanjau et al. [31] reported positive effects of physical activity on anxiety, in addition to the effect on depression. The COVIDsmart study may have encountered some external factors not included in the analysis that may confound the anxiety outcomes.

With social media becoming more ubiquitous in maintaining social relationships, researchers have been investigating its use and its potential impact on mental health in both the short- and long-term. Several studies have examined the relationship between social media use and depression and anxiety among adults [8, 32–34]. A meta-analysis by Lee et al. [35] found that overall, increased social media use (i.e., Facebook, Twitter, and Instagram) was linked to anxiety and depressive symptoms among young adults. This meta-analysis reported that COVID-19 may exacerbate existing mental health disorders that cause depression and anxiety among young adults [35]. In contrast, the middle-aged adults in the COVIDsmart study reported either an increase or decrease in social media use had higher depression (PHQ-9) and anxiety (GAD-7) scores compared to those whose use remained relatively stable. These findings are inconclusive but consistent with a non-COVID-19 related systematic review, which reported mixed results regarding the association between social media use and depression or anxiety among emerging adults and adolescents [8]. However, a systematic review by Karim et al. [8] identified a few studies, that focused on either adolescents, young adults, or adults, that did not find a significant association between increased social media use and mental health issues. Nevertheless, they also reported that many other studies did report any positive association [8]. One study suggested that social media use could be a risk factor for emotional dysregulation and perceived stress, but also a coping tool for dealing with mental health crises [34]. However, this study once again focused only on adolescents and young adults. Overall, the present study and the existing literature have reported conflicting results regarding the impact of social media use on mental health. Additional research using validated tools is necessary to identify which aspects and duration of social media use, specifically among middle-aged and older adults, and what are the risk or protective factors.

However, health behaviors rarely occur in a vacuum and are often products of other stressors such as loneliness and SE factors. The COVIDsmart study found that participants who reported an increased number of SE hardships as well as loneliness were also more likely to report higher depression (PHQ-9) and anxiety (GAD-7) scores. These results support the current literature that lonely individuals are less likely to improve health behaviors in such as tobacco and alcohol cessation, and additionally, loneliness has been linked to poorer mental health [36, 37]. Similarly, environmental stressors such as poor financial stability and poor health behaviors are interlinked where tobacco and alcohol consumption are often seen as coping mechanisms for stress and psychological distress [38, 39]. Social media use can have a varying impact of mental health where decreasing usage can exacerbate loneliness for individuals who struggle with maintaining online relationships, whereas increasing usage could also mitigate loneliness [40, 41]. However, Hampton et al. [40] also described that an increase in social media usage can introduce additional stressors such as reading negative news articles. Additionally, changes in health behaviors over time may be due to environmental changes at the personal, family, community, or policy levels, for example, losing a job, health status of family members, or community and national restrictions due to the COVID-19 pandemic.

Given that certain health behaviors, namely tobacco use, physical activity, and social media, are linked with an individual’s mental health, there should be a concerted effort to better disseminate accessible resources to help maintain positive health behaviors. Public health and community leaders should encourage individuals to access the many free resources online that can help stave off tobacco use, maintain physical activity, and manage healthy social media usage [42–46]. Additionally, these individuals can also utilize these on how to cope with stress using information from the Centers of Disease Control and Prevention or from the National Alliance on Mental Illness [47, 48]. Not only are healthy behaviors linked to better mental health outcomes, they also improve physical health, extend life expectancy, reduce risk of morbidity later in life, and gain financial stability [49].

The COVIDsmart study has a few strengths; no other studies have observed participants over a course of time to measure the longitudinal effect of health behaviors on mental health during the COVID-19 pandemic. Additionally, this HIPAA compliant online platform allowed for this study to occur during the social restrictions of the pandemic. However, it is also important to acknowledge its limitations. Firstly, the data collected relied on self-reported measures of health behaviors, depression, and anxiety, which may be influenced by reporting biases. Secondly, although multiple recruitment strategies were utilized to capture racial and ethnic minorities and vulnerable populations with low socioeconomic status, most participants were white, higher income, and highly educated with at least a Master’s degree [15]. The lack of racial and ethnic diversity and socioeconomic backgrounds limits this study, however, this is a common limitation with digital studies, as observed in other research studies [50]. Therefore, future studies should aim to capture a more diverse population. Thirdly, the study experienced a high rate of loss to follow-up, which may have been influenced by numerous factors such as decreased concern with COVID-19 following the easement of restrictions and availability of vaccines in 2021. Finally, the study's questions did not concretely measure changes in health behaviors such as the number of packs of cigarettes smoked, alcoholic beverages consumed, or the duration of physical activities done in the past two weeks, and therefore, future studies should aim to include discrete measurements when recording changes of health behaviors.

## Conclusions

The COVIDsmart study reveals that changes in health behaviors such as increased alcohol consumption, decreased physical activity, and social media use were associated with higher depression and/or anxiety scores among Virginians. These negative changes in behavior could be a response to the unprecedented environmental, social, and economic stressors caused by the pandemic. Continual monitoring of the effect of health behaviors on mental health is necessary to better understand the indirect consequences of the pandemic on mental health. These results have the potential to assist public health leaders to better understand behavioral changes during a pandemic to better tailor population-based health approaches to promote healthy behaviors. Adopting these healthy behaviors will promote better mental and physical health outcomes now and later in life.

### Supplementary Information

**Additional file 1: Appendix A. **Social and economic hardships questions and responses at baseline and six follow-ups. **Appendix B.** Questions and responses on the changes in alcohol consumption, tobacco consumption, physical activity, and social media use.

## Data Availability

The dataset generated and/or analyzed during the current study are not publicly available due to the data governance agreement between us and our partners, GMU and Vibrent Health Inc but are available from the corresponding author, SD, on reasonable request.

## Abbreviations

SE: Social and economic
HIPAA: Health Insurance Portability and Accountability Act
PHQ-9: Patient Health Questionnaire-9
GAD-7: Generalized Anxiety Disorder-7
N/A: Not applicable
MMRM: Mixed Model(s) with Repeated Measures
DSCF: Dwass, Steel, Critchlow-Fligner
