# Supplementary material for: Exploring the influence of behavioral factors on depression and anxiety scores during the COVID-19 pandemic: insights from the Virginia statewide COVIDsmart longitudinal study
Source: BMC Public Health. 2023 Sep 8;23:1749. doi: 10.1186/s12889-023-16614-7 (PMC10485957; doi:10.1186/s12889-023-16614-7)
Supplement: Supplementary file 1 — Additional file 1: Appendix A. Social and economic hardships questions and responses at baseline and six follow-ups. Appendix B. Questions and responses on the changes in alcohol consumption, tobacco consumption, physical activity, and social media use. [file 12889_2023_16614_MOESM1_ESM.docx]

Appendix A

Social and economic hardships questions and responses at baseline and six follow-ups

| Question at baseline | Question at monthly follow-ups | Responses |
| --- | --- | --- |
| Since the beginning of March 2020, have you or a member of your household experienced any of the following because of the spread of the coronavirus, or not? | Over the past 30 days, have you or a member of your household experienced any of the following because of the spread of the coronavirus, or not? | Lost income from a job or business |
|  |  | Lost money in retirement accounts or investments |
|  |  | Had your work hours reduced |
|  |  | Lost a job |
|  | Please select all that apply. | Filed for unemployment benefits |
| Please select all that apply. |  | Been unable to get groceries |
|  |  | Been unable to get disinfectant products (e.g., disinfecting wipes, hand sanitizer) |
|  |  | Been unable to get prescription medicine |
|  |  | Had trouble sleeping |
|  |  | Been unable to get adequate medical care |
|  |  | Been unable to visit family members who are at higher risk of infection |
|  |  | None of the above |

Appendix B

Questions and responses on the changes in alcohol consumption, tobacco consumption, physical activity, and social media use

| Question at baseline | Question at monthly follow-ups | | Responses | |
| --- | --- | --- | --- | --- |
| How have the following changed compared to this time last year before the COVID-19 pandemic affected the U.S.? | How have the following changed over the past 14 days? | | Four categorical choices | |
| Consumption of alcoholic beverages | | Increased | |  |
| Consumption of tobacco products, cigarettes, electronic cigarettes (ecigs, vapes, etc.) | | Decreased | |  |
| Physical activity | | Approximately the same | |  |
| Use of social media | | Not applicable | |  |
